# The prediction of the porcine pre-microRNAs in genome-wide based on support vector machine (SVM) and homology searching

**DOI:** 10.1186/1471-2164-13-729

**Published:** 2012-12-27

**Authors:** Zhen Wang, Kan He, Qishan Wang, Yumei Yang, Yuchun Pan

**Affiliations:** 1 School of Agriculture and Biology, Department of Animal Science, Shanghai Jiao Tong University, Shanghai, 200240, PR China; 2 Shanghai Key Laboratory of Veterinary Biotechnology, Shanghai, 200240, PR China; 3 Department of Biology, Faculty of Science, Hong Kong Baptist University, Hong Kong, China

**Keywords:** Porcine, Pre-miRNA, SVM, Homology searching

## Abstract

**Background:**

MicroRNAs (miRNAs) are a class of small non-coding RNAs that regulate gene expression by targeting mRNAs for translation repression or mRNA degradation. Although many miRNAs have been discovered and studied in human and mouse, few studies focused on porcine miRNAs, especially in genome wide.

**Results:**

Here, we adopted computational approaches including support vector machine (SVM) and homology searching to make a global scanning on the pre-miRNAs of pigs. In our study, we built the SVM-based porcine pre-miRNAs classifier with a sensitivity of 100%, a specificity of 91.2% and a total prediction accuracy of 95.6%, respectively. Moreover, 2204 novel porcine pre-miRNA candidates were found by using SVM-based pre-miRNAs classifier. Besides, 116 porcine pre-miRNA candidates were detected by homology searching.

**Conclusions:**

We identified the porcine pre-miRNA in genome-wide through computational approaches by utilizing the data sets of pigs and set up the porcine pre-miRNAs library which may provide us a global scanning on the pre-miRNAs of pigs in genome level and would benefit subsequent experimental research on porcine miRNA functional and expression analysis.

## Background

MicroRNAs (miRNAs) are a family of ~22nt endogenous non-coding RNAs [1,2]. Mature miRNAs are usually cleaved from ~90nt miRNA precursors (pre-miRNAs) which are derived from processing of a long primary miRNA (pri-miRNA) by a ribonucluease [3]. Increasing evidences have shown that miRNAs play fundamentally important roles in various biological processes, including cell proliferation [4-7], development timing [8,9], apoptosis [10,11], carcinogenesis [12-14], and response to different environmental stresses containing disease [15-17].

Since the first lin-4 miRNA of *C. elegans* was discovered in 1992 [18], more than 19000 miRNAs have been found in animals and plants. Currently, the miRNA Registry Database (Release 17, April 2011; http://mirbase.org), a comprehensive and searchable database of published miRNA sequences, contains 16772 entries representing hairpin pre-miRNAs, expressing 19724 mature miRNA products, in 153 species [19]. However, only 228 pre-miRNAs of pigs are included in this database, the number is far less than it really has.

Pre-miRNAs have similar hairpin-shaped stem loop structure, high minimal folding free energy index, and high evolutionary conservation. They become the important features which could be used in the computational identification of pre-miRNA [20-22]. To date, computational prediction has been broadly used to identify potential pre-miRNAs in animals and plants [23-25], because it is not limited by tissue specificity and time of miRNA expression. Especially, machine learning approaches such as random forest (RF) [26], naïve Bayes classifier [27], hidden Markov model [28,29] and SVM [30-32] have been adopted.

Although previous studies have identified a certain number of porcine pre-miRNAs, few researches in computational identification of pre-miRNAs based on the whole genome sequences are being done. Furthermore, most of the machine learning approaches are based on the data sets of human, while the features of the pre-miRNAs also exhibit the species-specificity. Therefore, we are aimed to identify the porcine pre-miRNA in genome-wide through computational approaches by utilizing the data sets of pigs in our study, which may provide us a global scanning on the pre-miRNAs of pigs in genome level. In our study, we built the SVM-based porcine pre-miRNAs classifier with a sensitivity of 100%, a specificity of 91.2% and a total prediction accuracy of 95.6%, respectively. As a result, 2204 and 116 porcine pre-miRNA candidates were separately detected by using SVM-based pre-miRNAs classifier and homology searching.

## Results and discussion

### Performance of the SVM-based pre-miRNAs classifier

**Figure 1:**
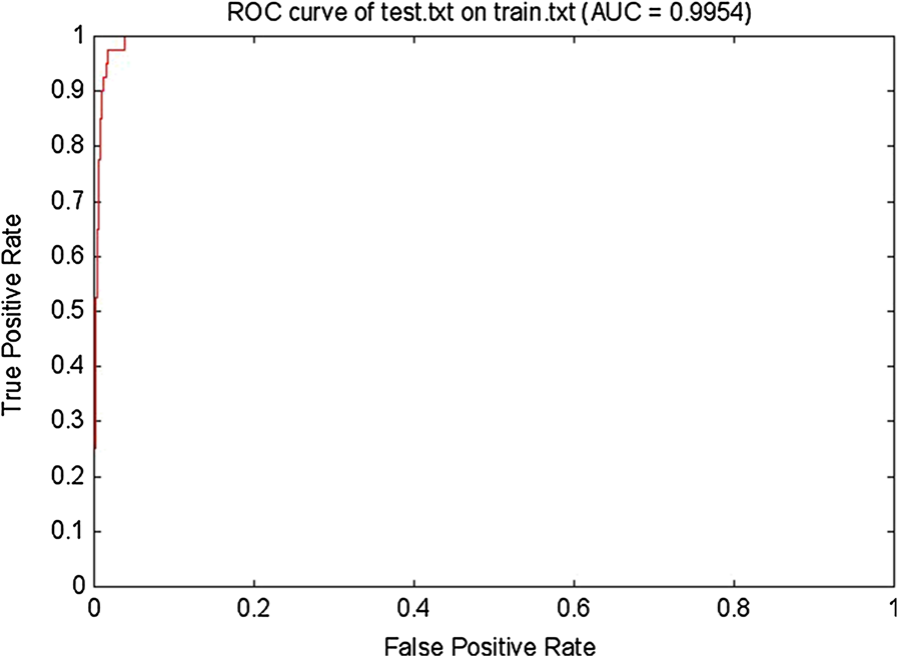
**ROC curve for the pre**-**miRNAs classifier on the test set.** The curve with more areas has better performance of the classifier. It showed the classifier reached a well performance.

SVM-based porcine pre-miRNAs classifier was built by using the data sets of pigs. Interestingly, all of porcine pre-miRNAs of the test set were correctly detected by our classifier, which achieved a sensitivity (SE) of 100%, a specificity (SP) of 91.2% and a total prediction accuracy (ACC) of 95.6%, respectively. The power of the pre-miRNAs classifier was given in Table 1. Moreover, the performance of the classifier was also tested by a ROC curve. As shown in the Figure 1, the classifier achieved a five-fold cross-validation rate of 99.54%. In a word, it indicated that our classifier was available for the prediction of porcine pre-miRNAs. Additionally, it also demonstrated that the comprehensive use of the pre-miRNAs features of the secondary structure and sequence information was an effective strategy in pre-miRNAs prediction.

**Table 1:** **Performance of the pre**-**miRNAs classifier on test sets.**

**Test set**	**Type**	**Size**	**Accuracy (%)**
**TE**-**S1**	Real	40	100%
**TE**-**S2**	Pseudo	1000	91.20%

Test set represents positive and negative set used to test the power of the pre-miRNAs classifier. Type represents the classification of the test set. Size is the number of the real or pseudo pre-miRNAs contained in test set. Accuracy is the percentage of the real or pseudo correctly recognized by pre-miRNAs classifier.

Xue et al. obtained an accuracy of 90% by using a set of features combining the local contiguous structures with sequence information to distinct the pre-miRNAs with that of pseudo pre-miRNAs [30], and those features have been used by several other pre-miRNA predicting methods [26,31,33]. Their studies demonstrated that those features were effective in pre-miRNA prediction. Thus, we also adopted those features in our study. Later, Jiang et al. found that the predicting performance significantly increased by combining the minimum of free energy (MFE) of the secondary structure or p-value feature with the local contiguous triplet structure composition feature. Their results indicated that a comprehensive feature vector was able to extract more information of a primary sequence and reach a better prediction performance [26]. Our classifier was capable of achieving a well prediction performance with an accuracy of 95.6% may be due to the using of a combined feature vector, because additional seven features used in our study have been proved to be one part of the optimized features subset in pre-miRNAs prediction by Wang et al. [3].

### Identification of pre-miRNAs candidates on pig genome using the SVM-based classifier

**Figure 2:**
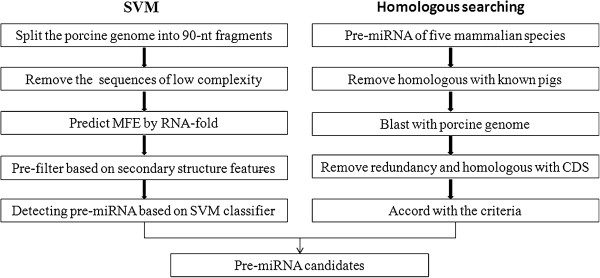
**Flowchart of the porcine pre**-**miRNA prediction procedure.**

Since the genome sequences contain the full information of a species and the database of non-coding RNA of pigs is quite incompletely, thus we used whole genome sequences to construct the prediction set (PR-S). After splitting the pig genome, we obtained more than 222 million short sequences. The PR-S constructed by short sequences passed by pre-filter was further distinguished by our SVM-based pre-miRNAs classifier. As pre-filter parameters would be very useful in filtering the pseudo pre-miRNAs from huge number of similar pre-miRNA sequences, those pre-filters were incorporated into the SVM-based classifier to predict novel pre-miRNAs. Except for the redundancy and the known pre-miRNAs, we finally got 2204 pre-miRNA candidates with the probability more than 0.99995 in the pig genome. They were formed into 1849 clusters according to their locations in genome wide (inter-distance <=50kb [34]). Those pre-miRNA candidates were blasted with porcine CDS and other non-coding RNA (NONCODE v3.0, http://www.noncode.org/NONCODERv3/). The result shown that 6 novel pre-miRNAs (coverage >90%, identities =100% with CDS) overlap with coding region. Namely, 2198 out of 2204 new pre-miRNAs are in the non-coding region. And none of pre-miRNAs (coverage >90%, identities >90% with non-coding RNAs) were found that overlap with other non-coding RNAs. The procedure for predicting porcine pre-miRNAs was given as Figure 2.

The large number of the novel pre-miRNA candidates indicated that there were still many unidentified pre-miRNAs in pigs. Previous studies estimated that the number of miRNAs have taken up to approximately 2-3% of the total number of genes in animal genomes [20]. According to our study, the number of the pre-miRNAs would be more than previous estimate. Expression profiling studies showed that most miRNAs were under the control of tissue-specific and development signaling, or both [35-37]. As a result, it may lead to a less number of miRNA identified by experimental methods and a low evaluate of pre-miRNAs’ number. Indeed, in our studies, we regarded those pre-miRNA candidates as the real porcine pre-miRNAs in the view of bioinformatics. Meanwhile, those pre-miRNA candidates were set up to the porcine pre-miRNA library, the detail information of which was given in Additional file 1.

To explore the location distribution of all the pre-miRNA candidates, we calculated the number of pre-miRNA candidates in each chromosome. And the chromosome 1 covered the maximum number of pre-miRNAs candidates, while the chromosome 18 included the minimum. To a large extent, the number was consistent with the length of chromosome, namely the bigger of the chromosome the more number of pre-miRNA candidates it contained. The density analysis of pre-miRNA in chromosome showed that chromosome X, 8 and 16 maintained the highest density of pre-miRNA. The chromosome 8 was also found that it had a high density of quantitative trait locus (QTL) (http://www.animalgenome.org/cgi-bin/QTLdb/SS/index). Thus, the result suggested other researchers should pay more attention to study the chromosome 8 of pigs in the future. The result of density analysis of pre-miRNA and QTL in chromosome was given in Additional file 2.

At the same time, 215 unique pre-miRNAs were identified in pigs by Solexa sequencing in another published study [38]. Based on the comparison this data with ours, we found that 49 (coverage >90%, identities >90% with predicting pre-miRNAs ) of above 215 unique pre-miRNAs were included in our study. In Chen et al.’s study, it mainly focused on identifying miRNAs in porcine backfat tissues. Tissues-specificity may lead to a bias on much more number of miRNAs identified in backfat tissues in their study, meanwhile some of their candidate miRNAs were unidentified by our method due to a limited length of 90-nt changed their features in our study. These may count for the low overlap rate. However, the result of Chen et al.’s study may still provide a piece of experimental evidence for our study. After the step of pre-filtering, a total of 160 known pre-miRNAs were retained in PR-S. 181 sequence fragments (coverage >90%, identities =100% with known pre-miRNAs) represented 115 known pre-miRNAs were detected by classifier. Namely, the sequence fragments of the known pre-miRNAs in the PR-S could be detected with the coverage of 72% (115 out of 160). The details those known pre-miRNAs sequence fragments were given in Additional file 3. There are several possible reasons accounting for that not all the reported porcine pre-miRNAs in miRNA Registry Database were covered in our studies. Firstly, not all the pre-miRNA sequences are expressed in the order of the genome sequence due to the RNA editing [39,40] , such as mir-381,mir-1271. According to our observation, 184 out of known 224 pre-miRNAs are completely identical to the sequence of the genome, thus 40 known pre-miRNA sequences unmapped to the genomic sequence data were filtered. Secondly, in order to reduce the pseudo pre-miRNAs as more as possible, the pre-filter parameters setting is up to some reported pre-miRNAs, such as the value of the minimal folding free energy index (MFEI). 160 out of 184 known pre-miRNAs were retained (20 known pre-miRNA were missed) after this step. Thirdly, the length of the short sequence is limited to 90-nt, while some features of pre-miRNAs (such as adjusted minimal folding free energy (N(AMFE)) and the adjust number of paired nucleotides (N(ANNB)) have connection with the sequence length [32,41], which may influence the features of 45 reported pre-miRNAs and lead them to be undetected.

Although the classifier produced a specificity of 91.2%, the candidate hairpins could be lead to a certain number of false positives in genome-wide prediction. Thus, the next problem removing those pseudo pre-miRNAs in the library is needed to be considered deeply.

### Identification of the pre-miRNAs candidates using the homologous searching

Since the pre-miRNA candidate sequences were split from genome with a specified length of 90-nt which may lead some of them undetected by our SVM classifier and the coverage of some model species with our SVM-based classifier result (coverage >85%, identities >85% with model species known pre-miRNAs) were 8% (human), 12% (mouse), 22% (rat), 16% (cow) and 31% (dog), which was not so high. The SVM-based classifier’s training set was composed by the porcine known pre-miRNAs to predict the novel pre-miRNAs of pigs. The feature of pre-miRNAs exhibits the species-specificity. It may cause our SVM-based classifier have some biases to detect more pre-miRNA possessed only by pigs. The species-specificity and homologous porcine pre-miRNAs unidentified in model species may contribute to the low overlap rate. It was necessary to make it up by some other computational methods. At present, besides the SVM classifier the homologous searching is also a widely used method for identifying the pre-miRNAs, because the pre-miRNAs have a highly conservation among the different species [20]. What’s more, in recent years, a large number of new pre-miRNAs were identified in some model species, such as Mouse, Human. Up to now, according to the records of miRNA Registry Database (Release 17, April 2011; http://mirbase.org), it contains human (1424), mouse (720), rat (408), cow (662) and dog (323). While, there are only 228 pre-miRNAs in pig. Therefore, it is quite necessary for us to do a homologous searching once again to find the new pre-miRNAs of porcine by using the identified pre-miRNAs in the other species.

According to the criteria mentioned in homologous searching method, we found 116 new pre-miRNAs candidates, and the detail information of which was given in Additional file 4. Interestingly, some pre-miRNAs candidates were mapped to more than one location of chromosomes. Guo et al. thought that cross-mapping events in pre-miRNAs revealed potential miRNA-mimics and evolutionary implications [42]. The newly identified porcine pre-miRNAs candidates belong to different miRNA families, such as miR-1282, miR-3059, miR-3120, miR-3618. Among them, miR-3120 initially identified from melanoma [43] and miR-3618 from human cervical cancer and normal cervices [44] have a highly conservation with pigs. We have also compared this result with the SVM-based and found no overlap between them. Actually, there were some of them passing SVM model before filtering in our study. However, when the prediction probability was set as more than 0.99995 to reduce false positive, they were filtered out with a result of no overlap between homology search and SVM-model candidates. There is no doubt that the high conservation of pre-miRNAs among the species also provides us a rapid way to identify the pig pre-miRNAs. This would be helpful to further enrich the resource of pre-miRNAs databases.

## Conclusions

In conclusion, we built the SVM-based pre-miRNAs classifier using the known pre-miRNAs and CDS sets of the pigs. From the porcine genome, we discovered 2204 new pre-miRNAs candidates by our SVM-based classifier and 116 pre-miRNAs candidates by homology searching. Our study would provide guidance on further experimentally verifying swine pre-miRNA in the future and offer the opportunity to research gene function and the genetic mechanism of complex traits in genome level.

## Methods

### Sequence data collection

The porcine genomic sequences were available from UCSC database (Mar 2010, http://hgdownload.cse.ucsc.edu/goldenPath/susScr2/bigZips/). The precursor sequences of known miRNAs of *Homo sapiens* (human), *Mus musculus* (mouse), *Rattus norvegicus* (rat), *Bos Taurus* (cow), *Canis familiaris* (dog) and *Sus scrofa* (pig) were obtained from miRNA Registry Database (Release 17, April 2011; http://mirbase.org) [19]. The porcine protein coding regions sequences (CDS) were downloaded from NCBI (ftp://ftp.ncbi.nih.gov/genomes/Sus_scrofa/RNA/), which were used as the pseudo pre-miRNA data.

### The length of the pre-miRNAs sequences (LS)

The statistical length distribution of porcine pre-miRNA from miRNA Registry Database is that 86% of them within 75~105 nt. In our study, both the porcine genome sequences and CDS were divided into short sequences using a 90-nt sliding window with 9-nt increments at one time [3,33].

### The complexity of the sequences

Low-complexity of the sequences, such as those with single nucleotide repeated > 8 times (for example, AAAAAAAA), dinucleotides repeated > 7 times (for example, AGAGAGAGAGAGAG), trinucleotides repeated > 4 times (for example, ATGATGATGATG), were removed for further analysis, since we observed few known pre-miRNA possessed such sequences. Additionally, the sequences with the region of gap were removed.

### MFE feature

MFE of the secondary structure was predicted by the Vienna RNA software package (RNAfold) (Version 1.8.5; http://www.tbi.univie. ac.at/~ivo/ RNA/) [45,46]. Previous studies indicated that pre-miRNAs have a high negative MFE and MFEI, which is a useful criterion to distinguish pre-miRNAs from all coding or non-coding RNAs [41]. The MFEI was calculated by the equation: MFEI = (- 100 × MFE/LS)/(G + C).

The three characteristics related to MFE were used as the feature vectors in SVM, and they were defined as follows:

(1) $$N\left(\mathrm{MFE}\right)=\left(-\mathrm{MFE}\right)/1000$$

(2) $$N\left(\mathrm{MFE}\right)=\mathrm{MFEI}/10$$

(3) $$N\left(\mathrm{AMFE}\right)=\left(-\mathrm{MFE}\right)/\left(10\times \mathrm{LS}\right)$$

### Base-pairings and the secondary structure features

Because nucleic acid G can be paired with C or U, the base-pairings on the stem of the hairpin structure included the GU wobble pairs. And the threshold of the minimum base-parings of real pre-miRNA was 18. Indeed, the stem of the hairpin structure is highly conserved in pre-miRNAs, so we still only considered the stem regions of the pre-miRNA. The number of paired nucleotides (NNB), the adjust number of paired nucleotides (ANNB) and the number of nucleotides of the stem parts (NNS) were utilized as three feature vectors, defined as follows:

(4) $$N\left(\mathrm{NNB}\right)=\mathrm{NNB}/1000$$

(5) $$N\left(\mathrm{ANNB}\right)=\mathrm{NNB}/\mathrm{LS}$$

(6) $$N\left(\mathrm{NNS}\right)=\mathrm{NNB}/\mathrm{NNS}$$

Meanwhile, we denoted the contents of GC as follows:

(7) $$N\left(\mathrm{GC}\right)=\mathrm{GC}/1000$$

Besides, seven other features, including the structural diversity (N(Diversity)) (8), the frequency of the MFE structure (N(Freq/100)) (9) [46], adjusted base pair distance (N(dD)) (10) [47], average distance between internal loops (N(D_interlp/1000)) (11), the ratio of |A-U| to the length of sequence (N(|A-U|/LS)) (12), the length of the longest relaxed symmetry region (N(l_rsym_rgn/100)) (13) and the length of the longest symmetry region (N(l_sym_rgn/100)) (14), which were found as the optimized features for pre-miRNAs prediction according to the studies of Wang et.al [3], were also adopted.

### The local adjacent sequence-structure features

Previous studies have shown that local sequence features play a crucial role in pre-miRNAs [48]. Additionally, Xue et al. found that the distributions of local contiguous sub-structures of pre-miRNAs are significantly distinguished with that of pseudo pre-miRNAs [30]. Therefore, in our study, we also characterized the secondary structure of pre-miRNAs by combining of the sequence information with the local contiguous structures.

There are only two conditions for each nucleotide in the predicted secondary structure by RNAfold [45], paired or unpaired, denoted by brackets “(” and dots “.”, respectively. The left bracket “(” represents that paired nucleotide located near 5′-end which can be paired with another nucleotide at the 3′-end indicated by a right bracket “)”. We used “(” for both situations without differentiating “(” or “)”, because no evidence has indicated that mature miRNAs have a preference of the 3′ or 5′ arms of their hairpin precursors. Obviously, for any 3 adjacent nucleotides, there are eight possible structure units: “(((”, “((.”, “(.(”, “.((”, “(.”, “.(. ”, “.(”, “…”. Furthermore, by considering the left nucleotide among the three [31], there are 32 possible sequence-structure units, left-triplet coding ,denoted as “A(((”, “U(((”, “A((.”, etc. as shown in Additional file 5[31]. Here, we only considered the stem regions of a pre-miRNA by excluding the external single-stranded parts and the terminal loop. Similar features have been adopt by pioneer work, e.g. that Zhao et al. [31]. The frequency of each left-triplet coding of pre-miRNA was counted to create the 32 feature vectors. After normalizing, the frequency was used as input features for SVM. Combining with 14 features above, in all 46 feature vectors (summarized in Additional file 6) were taken as the input of SVM.

### The pre-filter parameters of secondary structure features

Each sequence secondary structure, predicted by the Vienna RNAfold, was passed through a set of filter parameters. The filtering parameters [33,49,50]related to some terms of secondary structures were given as Additional file 7[3], which were shown below.

(a) The number of hairpin loops = 1;

(b) The number of symmetrical loops < 6.

(c) The number of asymmetrical loops < 4.

(d) The number of bulges < 5.

(e) The total number of symmetrical and asymmetrical loops < 8.

(f) The total number of symmetrical, asymmetrical loops and bulges <10.

(g) The number of the base pairing >17.

(h) The value of ANNB is between 0.3~0.43.

(i) The length of symmetrical loops < 5.

(j) The length of asymmetrical loops <6.

(k) The length of bulges < 6.

(l) The MFE < −15kal/mol.

(m) The MFEI >0.7.

(n) The percentage of the GC contents is between 30-70%.

### SVM data set

Among the 228 known porcine pre-miRNAs, whose secondary structures with no multiple loops were considered. 224 pre-miRNAs, covering more than 98% of all the reported porcine pre-miRNAs, were retained. We randomly extracted 184 pre-miRNAs from them as one part of training set (TR-S) and the remaining 40 pre-miRNAs formed into the test set 1 (TE-S1).

A pseudo pre-miRNAs set was collected from the porcine CDS and 5677 pseudo pre-miRNAs were selected due to their similar stem-loop structures to real pre-miRNAs. The criteria for extracting the pseudo pre-miNRAs from CDS segment was complied with the pre-filter parameters of the secondary structure features above. 184 pseudo pre-miRNAs selected randomly from the pseudo pre-miRNAs set composed another part of TR-S. Furthermore, we randomly took out 1000 pseudo pre-miRNA from the remaining pseudo pre-miRNAs set as test set 2 (TE-S2).

**Figure 3:**
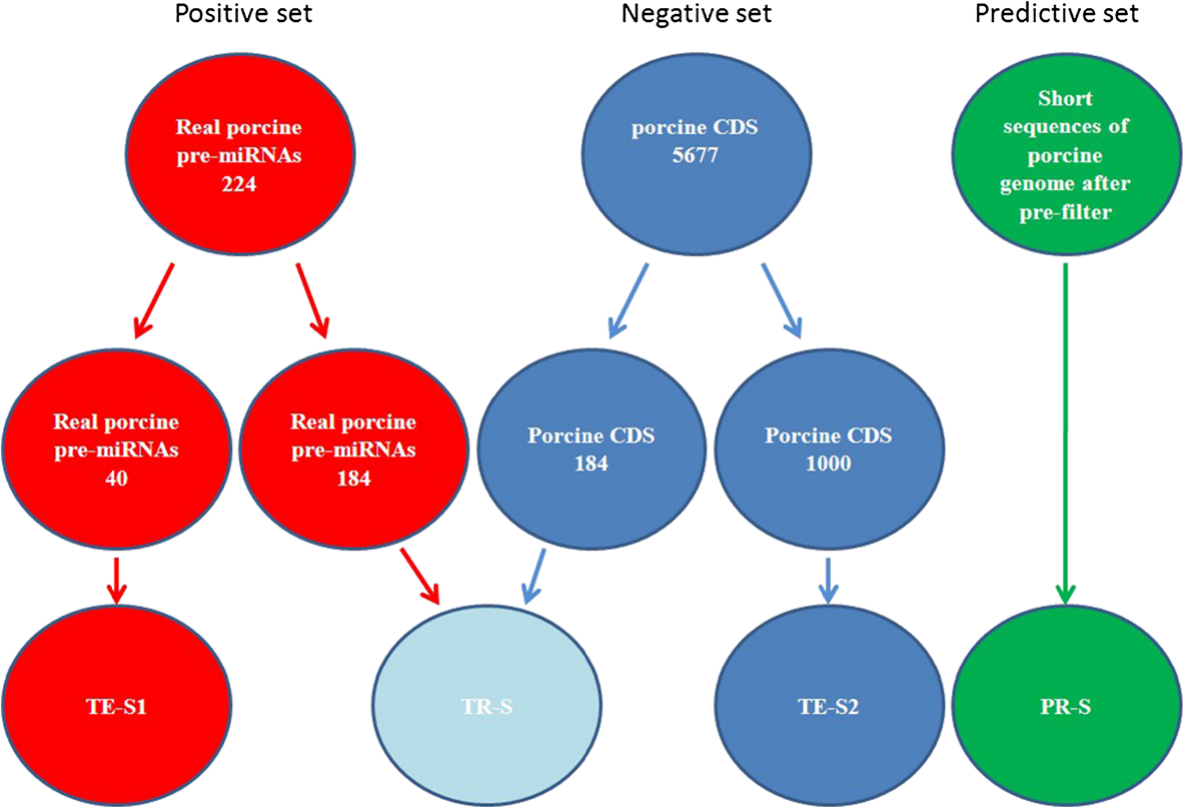
**The composition of each set including the training set (TR**-**S), testing set (TE**-**S1 and TE**-**S2) and predictive set (PR**-**S).** 184 real and pseudo porcine pre-miRNAs are randomly extracted from positive set (224 known real porcine pre-miRNAs) and negative set (5677 porcine CDS), respectively, and then they form into the training set. The remaining 40 real porcine pre-miRNAs compose the test set 1 (TE-S1). 1000 pseudo pre-miRNAs from the remaining negative set are randomly selected as test set 2 (TE-S2). Both TE-S1 and TE-S2 are used to test the performance of the SVM-based pre-miRNAs classifier. The predicting set (PR-S) is constructed by the porcine genome sequence fragments passed the pre-filter parameters of secondary structure features.

In addition, the porcine genome sequence fragments split from genome using a 90-nt sliding window with 9-nt increments at one time, passed the pre-filter parameters of secondary structure features (including (a),(g),(l) and (MFEI>0.6)), were collected for further identifying by SVM classifier and constructed the PR-S. The composition of each set was shown in Figure 3.

### SVM

SVM, based on statistical theory [51], has a good generalization ability [52]. Therefore, in our study, SVM was adopted as a classifier to identify the real and pseudo pre-miRNAs. It was trained by the TR-S with the performance estimated by TE-S and applied to the PR-S. A 46-dimension feature vector referred to the above was taken as the input of SVM and the output was the number value “1”, which means the true, or “-1” indicating the false.

In our study, we downloaded a widely used software package Libsvm (Version 3.1, April 2011; http://www.csie.ntu.edu.tw/~cjlin/libsvm/)[53] to carry out our work. In order to acquire SVM classifier with optimal performance, we applied five cross-validation in model training, which could obtain the optimal penalty parameter C and the RBF kernel parameter g. Meanwhile, the performance of the SVM classifier was evaluated by following the assessment system used in RF [26].

### Homologous searching

We chose pre-miRNAs of five other mammalian species (including human, mouse, rat, cow and dog), which have a highly homology with pigs. Firstly, we removed the pre-miRNAs which have a highly homologous with 228 known porcine pre-miRNAs from the total pre-miRNAs of five species by utilizing the software of BLAST (ncbi-blast-2.2.25+; ftp://ftp.ncbi.nlm.nih.gov/blast/executables/blast+/LATEST/)[54]. Next, the remaining pre-miRNAs were blasted with the genome sequence of pigs and the sequence fragments (coverage >85%, identities >85% with pre-miRNAs) were retrieved from genome. Lastly, after discarding the redundant sequences, the sequences were regarded as pre-miRNA candidates if they accorded with the following criteria [55,56]:(i) an RNA sequence can fold into an stem-loop hairpin structure;(ii) predicted secondary structures had MFE less than -15kcal/mol;(iii) minimum base pairings on the stem of the hairpin structure is18;(iv) no multiple loops; (v) the GC contents is between 30~70%.

## Competing interests

The authors declare that they had no competing interests.

## Authors’ contributions

YP,QW and ZW designed the study. ZW collected the datasets from databases and analyzed the data, then prepared the original draft the manuscript. KH and YY guided the SVM analysis and the interpretation of the results. YP and QW reviewed the manuscript. All authors read and approved the final manuscript.

## Supplementary Material

Additional file 1 **The list of porcine pre**-**miRNA candidates predicted by SVM**-**based classifier.** The data provided represent the list of porcine pre-miRNA candidates predicted by SVM-based classifier in the whole genome of the pigs, and containing the information of their length, location in chromosome and genome location clusters. Click here for file

Additional file 2 **The result of density analysis of pre**-**miRNA and QTL in chromosome.** The data provided the information of the number of pre-miRNA and QTL and their density in each chromosome. Click here for file

Additional file 3 **The list of porcine known pre**-**miRNA fragments of 90nt detected by SVM**-**based classifier.** The data provided represents the list of porcine known pre-miRNA detected by SVM-based classifier in the whole genome of the pigs, and containing the information of their length, location in chromosome and the name of the represented known pre-miRNA. Click here for file

Additional file 4 **The list of porcine pre**-**miRNA candidates predicted by homology searching.** The data provided represent the list of porcine pre-miRNA candidates predicted by homology searching, and containing the information of their length and location in chromosome. Click here for file

Additional file 5 **Local sequence**-**structure features of a hairpin were denoted by the left**-**triplet coding.** Left-triplet elements are used to represent the local structure sequence features of a hairpin. The nucleotide type at the left and three local continuous substructures compose the left-triplet element. The appearances of all 32 possible triplet elements are counted along a hairpin segment to form a 32-dimensional vector, which is normalized to be the input vector for SVM. Click here for file

Additional file 6 The 46 features used by SVM-based porcine pre-miRNAs classifier. Click here for file

Additional file 7 **The primary sequence of the has**-**let**-**7e precursor and the locations of some terms in the secondary structure.** The upper part gives the primary structure of has-let-7e and the lower one shows the secondary structure and the correlative terms with varied colors. Click here for file
