# Supplementary figures and images for: The prediction of the porcine pre-microRNAs in genome-wide based on support vector machine (SVM) and homology searching
Source: BMC Genomics. 2012 Dec 27;13:729. doi: 10.1186/1471-2164-13-729 (PMC3545972; doi:10.1186/1471-2164-13-729)

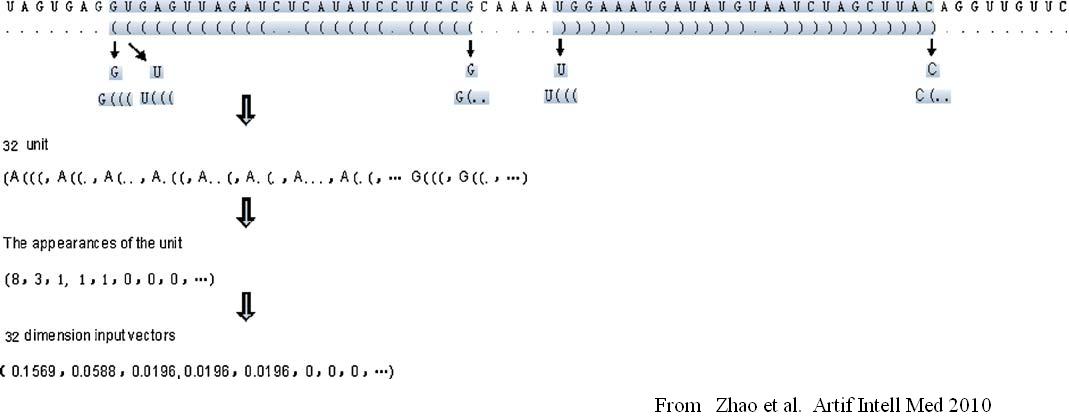

Supplement: Additional file 5 — Local sequence-structure features of a hairpin were denoted by the left-triplet coding. Left-triplet elements are used to represent the local structure sequence features of a hairpin. The nucleotide type at the left and three local continuous substructures compose the left-triplet element. The appearances of all 32 possible triplet elements are counted along a hairpin segment to form a 32-dimensional vector, which is normalized to be the input vector for SVM. [file 1471-2164-13-729-S5.jpeg]

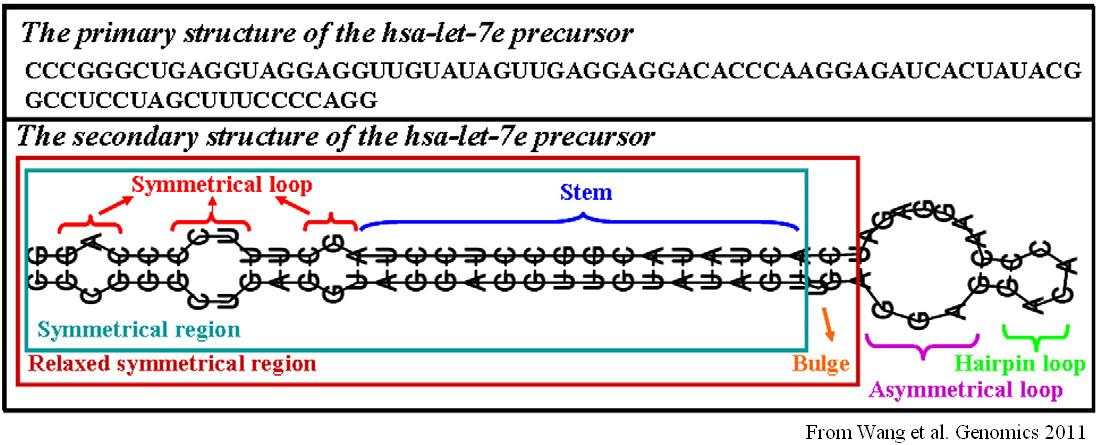

Supplement: Additional file 7 — The primary sequence of the has-let-7e precursor and the locations of some terms in the secondary structure. The upper part gives the primary structure of has-let-7e and the lower one shows the secondary structure and the correlative terms with varied colors. [file 1471-2164-13-729-S7.jpeg]
