# Supplementary material for: The prediction of the porcine pre-microRNAs in genome-wide based on support vector machine (SVM) and homology searching
Source: BMC Genomics. 2012 Dec 27;13:729. doi: 10.1186/1471-2164-13-729 (PMC3545972; doi:10.1186/1471-2164-13-729)
Supplement: Additional file 6 — The 46 features used by SVM-based porcine pre-miRNAs classifier. [file 1471-2164-13-729-S6.docx]

The 46 features used by SVM-based porcine pre-miRNAs classifier

| Feature | Description |
| --- | --- |
| The left-triplet coding | The frequency of each left-triplet coding of pre-miRNA (including 32 units) |
| N(MFE) | Minimal folding free energy |
| N(MFEI) | Minimal folding free energy index |
| N(AMFE) | Adjusted minimal folding free energy |
| N(NNB) | The number of paired nucleotides |
| N(ANNB) | The number of paired nucleotides |
| N(NNS) | The ratio of the number of paired nucleotides to the number of nucleotides of the stem parts |
| N(GC) | The content of GC |
| N(Diversity) | The structural diversity |
| N(Freq/100) | The frequency of the MFE structure |
| N(dD) | Adjusted base pair distance |
| N(D_interlp/1000) | Average distance between internal loops |
| N(\|A-U\|/LS) | The ratio of \|A-U\| to the length of sequence |
| N(l_rsym_rgn/100) | The length of the longest relaxed symmetry region |
| N(l_sym_rgn/100) | The length of the longest symmetry region |
